# Inhibition of an Erythrocyte Tyrosine Kinase with Imatinib Prevents *Plasmodium falciparum* Egress and Terminates Parasitemia

**DOI:** 10.1371/journal.pone.0164895

**Published:** 2016-10-21

**Authors:** Kristina R. Kesely, Antonella Pantaleo, Francesco M. Turrini, Peter Olupot-Olupot, Philip S. Low

**Affiliations:** 1 Purdue Institute for Drug Discovery, Purdue University, West Lafayette, 47907, United States of America; 2 Purdue Department of Chemistry, Purdue University, West Lafayette, 47907, United States of America; 3 Department of Biomedical Sciences, University of Sassari, Sassari, Italy; 4 Department of Genetics, Biology and Biochemistry, University of Turin, Turin, Italy; 5 Department of Paediatrics/Research Unit, Mbale Regional Referral Hospital, Mbale, Uganda; Liverpool School of Tropical Medicine, UNITED KINGDOM

## Abstract

With half of the world’s population at risk for malaria infection and with drug resistance on the rise, the search for mutation-resistant therapies has intensified. We report here a therapy for *Plasmodium falciparum* malaria that acts by inhibiting the phosphorylation of erythrocyte membrane band 3 by an erythrocyte tyrosine kinase. Because tyrosine phosphorylation of band 3 causes a destabilization of the erythrocyte membrane required for parasite egress, inhibition of the erythrocyte tyrosine kinase leads to parasite entrapment and termination of the infection. Moreover, because one of the kinase inhibitors to demonstrate antimalarial activity is imatinib, i.e. an FDA-approved drug authorized for use in children, translation of the therapy into the clinic will be facilitated. At a time when drug resistant strains of *P*. *falciparum* are emerging, a strategy that targets a host enzyme that cannot be mutated by the parasite should constitute a therapeutic mechanism that will retard evolution of resistance.

## Introduction

The inaugural meeting of the World Health Organization in 1948 designated malaria as one of six consensus global health priorities [1]. However, despite remarkable victories over smallpox (1979) and polio (1988), malaria still remains a major health problem, with nearly half of the world’s population at risk of contracting the disease and nearly 600,000 deaths per year arising from the parasitemia [1]. According to the WHO, children under the age of 5 are especially vulnerable, with one child dying of the disease every minute [2]. The absence of an effective vaccine [3] and the continuing emergence of resistance to existing antimalarials [4, 5] foreshadow a possible global health crisis that can only be addressed by introduction of mutation-resistant therapies [6, 7].

Five species of protozoans belonging to the genus *Plasmodium* cause malaria, but the most lethal of the species is *Plasmodium falciparum*. During the intra-erythrocytic phase of its life-cycle, the parasite matures through ring, trophozoite, and schizont stages that are characterized by increasing tyrosine phosphorylation of the erythrocyte membrane protein, band 3 [8–10]. Because this phosphorylation has been shown to dramatically weaken the membrane, leading to erythrocyte vesiculation and eventual red cell rupture [11, 12], the hypothesis has arisen that *P*. *falciparum* promotes tyrosine phosphorylation of band 3 in order to facilitate its egress from the erythrocyte, thereby enabling its propagation. In exploring this hypothesis, we learned that others have discovered that the *P*. *falciparum* genome surprisingly encodes no classical tyrosine kinase [13, 14], suggesting that any tyrosine phosphorylation of band 3 must either be performed by an erythrocyte tyrosine kinase or an unrelated *P*. *falciparum* kinase with the ability to phosphorylate tyrosines [15, 16]. This observation in turn lead us to believe that an inhibitor of the erythrocyte tyrosine kinase could disrupt the *P*. *falciparum* life cycle by preventing its egress from the red cell. To test this hypothesis, we screened inhibitors of the five known erythrocyte tyrosine kinases (i.e. syk [11, 17], lyn [18], hck [19], fgr [19], and src [20]) for their anti-malarial activity. In this paper, we report that imatinib, a well-tolerated tyrosine kinase inhibitor that is FDA-approved for use in children, prevents parasite-induced tyrosine phosphorylation of band 3 and terminates *P*. *falciparum* parasitemia in vitro by blocking parasite egress at clinically relevant concentrations.

## Results

### Tyrosine Kinase Inhibitor Treatment

The major erythrocyte membrane protein, band 3 (AE1, SLC4A1, anion transporter) forms the predominant bridge connecting the red cell membrane to its spectrin/actin cytoskeleton via an association with ankyrin [21–24]. In previous studies we have shown that tyrosine phosphorylation of band 3 causes dissociation of ankyrin [11, 25], leading to rupture of this membrane-to-cytoskeleton bridge and the consequent membrane destabilization, vesiculation and hemolysis [11, 12, 26, 27]. Curiously, *P*. *falciparum* infection of human erythrocytes (RBCs) promotes a gradual but significant increase in band 3 tyrosine phosphorylation [8] despite the absence of any tyrosine kinases encoded in the parasite genome [13, 14]. Because this gradual increase in band 3 tyrosine phosphorylation coincides with the rise in membrane vesiculation and eventual erythrocyte rupture [8], this observation raises the possibility that an erythrocyte tyrosine kinase might be co-opted by the parasite to promote erythrocyte membrane destabilization and facilitate merozoite egress. The purpose of the studies described below was to investigate this hypothesis.

To begin to assess which erythrocyte tyrosine kinase might be activated by *P*. *falciparum*, we examined the abilities of inhibitors of the five known erythrocyte tyrosine kinases to prevent parasite propagation in vitro. As shown in Table 1 and S2 Fig, untreated parasite cultures and cultures treated with dasatinib, i.e. a broad spectrum tyrosine kinase inhibitor (TKI) that suppresses the activities of bcr-abl, src, lck, yes, fyn, lyn, hck, c-kit, EPHA2, and PDGFRβ displayed an unabated increase in parasitemia, demonstrating that dasatinib can neither prevent parasite egress nor block *P*. *falciparum* reinvasion/proliferation in fresh human RBCs. In contrast, imatinib, PRT062607, gefitinib, R406, bafetinib, nilotinib, and PP-121 all showed measurable antimalarial activity, with the latter three displaying higher potencies than the former four (S1–S3 Figs). However, because imatinib i) was previously shown to inhibit the major erythrocyte tyrosine kinase (syk) that phosphorylates band 3 [28], ii) was the only inhibitor tested that is FDA-approved for use in children [29, 30], and iii) can be taken daily in perpetuity by cancer patients with little associated toxicity [31, 32], this tyrosine kinase inhibitor was selected for further investigation.

**Table 1:** Identification of erythrocyte tyrosine kinase inhibitors that treat *Plasmodium falciparum* malaria in vitro.

Drug Name	Kinase(s) Inhibited	Present in RBC?	Drug Status (FDA Approval)	Approved for Pediatric Use?	IC_50_ (μM)*
Bafetinib	Bcr-Abl	No	Phase I/II trials completed	No	1.34^a^^,^^b^
	Lyn	Yes			
Dasatinib	Bcr-Abl	No	Sprycel^®^ approved for leukemia in adults only.	No	>10^b^
	Src, Lck, Yes, Fyn	No			
	Lyn, Hck	Yes			
	c-kit	No			
	EPHA2	No			
	PDGFRβ	No			
R406	Syk	Yes	Phase II trial underway for ITP	No	>1, <10^b^
Gefitinib	EGFR	No	Iressa^®^ approved for NSCLC cancer in adults only.	No	>1, <10^b^
	Lyn	Yes			
Imatinib	Bcr-Abl	No	Gleevec^®^ approved for CML and GIST adults and children.	Yes	3.03^a^^,^^b^
	PDGFRβ	No			
	c-kit	No			
	SCF	No			
	Syk, Lyn	Yes			
Nilotinib	Bcr-Abl	No	Tasigna^®^ approved for CML in adults only.	No	<1^b^
	Lyn	Yes			
PP-121	Abl	No	Removed from Pfizer drug development pipeline	No	0.83^a^^,^^b^
	PDGFRβ	No			
	VEGFR2	No			
	Hck, Src	Yes			
PRT062607	Syk, Lyn, Hck, Fgr	Yes	Phase I trial completed	No	3.29^a^^,^^b^

*Approximate IC_50_ values for each drug were determined by treating ring stage cultures of *P*. *falciparum* with different concentrations of each drug and quantitating residual parasitemia 60 h later.

^a^ *P*. *falciparum* Palo Alto strain was used for the experiments

^b^ *P*. *falciparum* Dd2 strain was used for the experiments

### Effect of imatinib on malaria parasite maturation and propagation in vitro

**Fig 1:**
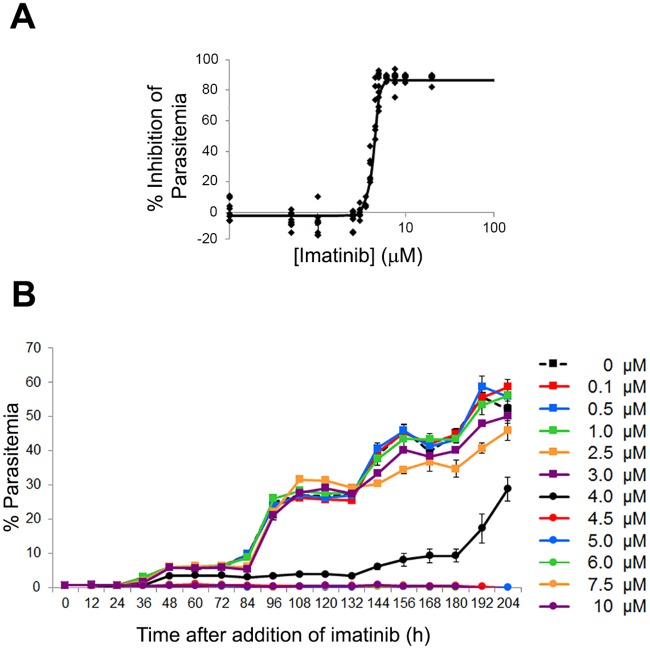
Effect of imatinib on survival of *P*. *falciparum* malaria in human erythrocyte cultures. (A) Effect of different concentrations of imatinib on the survival of *P*. *falciparum* in human erythrocyte cultures. Synchronized ring stage (12hpi) *P*. *falciparum* (Palo Alto strain) cultures at 0.5% parasitemia were treated with the indicated concentrations of imatinib, and after 60h incubation, % parasitemia was quantitated by flow cytometry (see Methods). Results were obtained from three independent experiments, with each concentration examined in triplicate in each experiment. (B) Effect of a single treatment of imatinib on the long term survival of *P*. *falciparum* in human erythrocyte cultures. Synchronized ring stage parasites were treated 12h post-infection (time 0) with increasing concentrations of imatinib and % parasitemia was evaluated every 12 hours, as described above. To assure the continuous availability of nutrients for any proliferating parasites and to prevent loss of parasitized RBCs during media replacement, 2/3 of the media was replaced with fresh media every 24h beginning at the 72-hour time point. Results were obtained with each concentration examined in triplicate.

To obtain an accurate assessment of the anti-malaria potency of imatinib in vitro, synchronized *P*. *falciparum* cultures were treated with increasing doses of imatinib and parasitemia was assessed 60 h after drug administration (i.e. 72 h after initiation of the first cycle). As shown in Fig 1A, imatinib effectively suppressed parasitemia in culture, with essentially complete inhibition achieved at ~5 μM. To verify that this suppression was stable and not a temporary interruption of parasite growth, synchronized cultures were treated with a single dose of imatinib and monitored through 5 consecutive infective life cycles. As seen in Fig 1B, untreated cultures showed a stepwise increase in parasitemia through all five life cycles, i.e. demonstrating that the parasites were capable of proliferating in the erythrocyte cultures throughout the 10-day experiment. In contrast, concentrations of imatinib that were previously found to eliminate all parasitemia by the end of the parasite’s first life cycle (Fig 1B) were also found to prevent parasitemia recurrence when cultures were monitored for an additional four life cycles. Although the cultures treated with 4 μM imatinib appeared to recover after the 132 hour time point, we suspect this delayed rise in parasitemia derives from the proliferation of surviving parasites not killed during initial exposure to this suboptimal concentration (4 μM) of imatinib rather than emergence of drug resistance. At higher imatinib concentrations the data suggest that drug exposure can completely suppress parasite proliferation (Fig 1B and S1 Table).

### Tyrosine phosphorylation of Band 3 in infected RBCs

**Fig 2:**
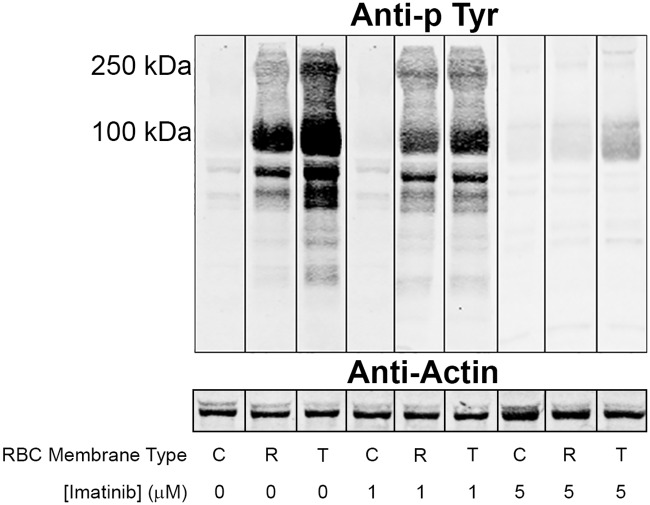
Effect of imatinib on tyrosine phosphorylation of band 3 in *P*. *falciparum* infected erythrocytes. Membranes were isolated from uninfected control erythrocytes (C) or *P*. *falciparum*-infected erythrocytes treated at the ring (R) or trophozoite (T) stage with the indicated concentrations of imatinib. Isolated membranes were separated by SDS-PAGE and transferred to nitrocellulose prior to western blotting with an anti-phosphotyrosine antibody. The tyrosine phosphorylated band at 100kDa is band 3, as demonstrated by mass spectrometry analysis of phosphopeptides [12]. Anti-actin staining of the same blots is presented as gel loading control.

We next determined whether imatinib might cause elimination of the parasitemia by inhibiting the parasite-induced tyrosine phosphorylation of band 3, as hypothesized in the proposed mechanism. As shown in Fig 2, anti-phosphotyrosine immunoblots of membranes derived from uninfected RBCs displayed no detectable tyrosine phosphorylation of band 3, consistent with previous observations by multiple authors [11, 33–35]. In contrast, membranes from ring and trophozoite stage infected RBCs showed significant tyrosine phosphorylation of band 3 that appeared to increase, as previously noted [9, 12], as maturation of the parasite proceeded (membranes from schizont stage RBCs were very difficult to isolate and consequently not examined). Importantly, membranes from infected RBCs treated with a low concentration of imatinib (1 μM) displayed reduced tyrosine phosphorylation of band 3 and this suppression of band 3 tyrosine phosphorylation was almost quantitative by 5 μM imatinib; i.e. the concentration that eliminated all parasitemia. These data demonstrate that imatinib inhibits the tyrosine phosphorylation of band 3 over the same concentration range where it inhibits parasitemia.

### Inhibition of parasite egress from infected RBCs

**Fig 3:**
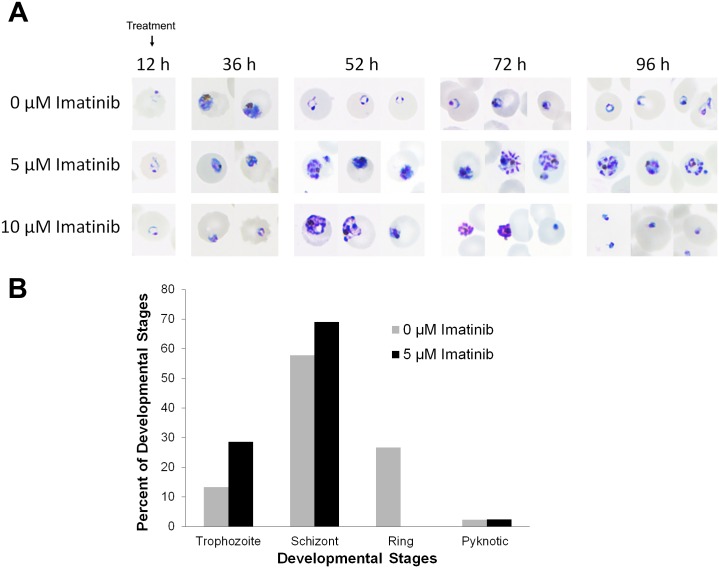
Effect of imatinib treatment on *P*. *falciparum* maturation and egress. Synchronized ring stage parasites (12 hours after parasite invasion) were either left untreated (top row) or treated with 5 μM (middle row) or 10 μM (bottom row) imatinib, and parasite morphology was assessed on blood smears by light microscopy at 12, 24, 36, 42, 44, 46, 48, 50, 52, 72, 96 hours after invasion using a Nikon Eclipse CI-L microscope, Plan Fluor 100X oil objective, Numerical Aperture 1.3 at room temperature equipped with a Nikon DS-Fi2 digital camera and NIS-Elements Basic Research acquisition software. (A) Representative images at the indicated time points are shown, focusing on morphology of individual infected cells to enable easier comparison. Note that at the 52 h time point, expected ring stage parasites are only seen in untreated cultures. (B) Quantitative analysis of 0 or 5μM imatinib treated malaria infected RBCs at 42 hpi.

The proposed mechanism of imatinib action further posits that inhibition of band 3 tyrosine phosphorylation should prevent the membrane destabilization required for merozoite egress at the end of the parasite’s life cycle. To test this step in the mechanism, we next monitored both the development and eventual escape of parasites from untreated and imatinib-treated synchronized *P*. *falciparum* cultures. As shown in the light micrographs of Fig 3A, healthy parasites from untreated cultures displayed the normal 48 h life cycle, proceeding from RBC infection through ring, trophozoite and schizont stages of development to merozoite egress and reinvasion within the expected time span (see images at 12, 36 and 52 h post infection). In contrast, 5 μM imatinib-treated cultures displayed an interruption in development, with early schizont-like parasites prominent at the 52 h time point when untreated cultures were beginning their next life cycle. Moreover, many infected erythrocytes were found to contain either dead or dying parasites. At the 72 h time point, the 5 μM imatinib-treated cultures were still dominated by either merozoite-like parasites entrapped within infected RBCs or pyknotic parasites displaying condensed DNA. By the beginning of the third life cycle (i.e. 96 h time point), a few merozoite-like structures still remained in the treated samples, but most of the infected cells contained disintegrating parasites with condensed DNA.

When the concentration of imatinib was increased to 10 μM, the trend of parasite entrapment followed by disintegration was still observed, however, additional indications of growth arrest and interruption of parasite maturation preceded parasite entrapment, suggesting that this higher dose was more generally toxic. Indeed, all of the parasitized cells appeared pyknotic by the 96 h time point, and few if any infected cells displayed signs of normal maturation at any time point. These data suggest that lower doses of imatinib may terminate parasitemia by prevention of parasite egress, whereas higher doses likely induce parasite death by additional unrelated mechanisms. A quantitative analysis of the fraction of infected RBCs at each stage of parasite development following treatment with either 0 or 5 μM imatinib is shown in Fig 3B. Importantly, at 42h post infection, when 28% of the untreated parasitized RBCs had egressed and progressed to the ring stage of their second life cycle, none of the infected RBCs treated with imatinib had progressed to this stage. Since analysis at other time points show a similar failure of imatinib-treated cultures to enter their second life cycle, these data suggest that exposure to imatinib inhibits progression of the parasites through their normal life cycle.

### Stage Specific Sensitivity

**Fig 4:**
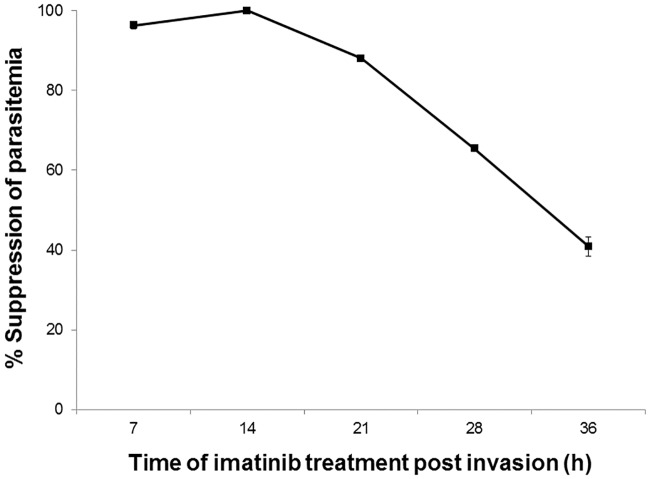
Sensitivity of *P*. *falciparum* cultures to imatinib at different stages of the parasite’s life cycle. Imatinib (8 μM) was added to synchronized *P*. *falciparum* (Dd2 strain) cultures at different stages of their life cycle (i.e., ring stage ~7hpi, late ring stage ~14 hpi, trophozoite stage ~21 hpi, late trophozoite stage ~28 hpi, and schizont stage ~36 hpi). Parasitemia was measured after the untreated parasites had progressed 24 h into their second infective cycle. The absence of detectable parasitemia is reported in the plot as 100% suppression. Note that imatinib must be present by late ring stage to achieve quantitative suppression of the parasitemia.

Patients with symptomatic malaria experience severe fever roughly every 48 h, demonstrating that the parasite’s life cycle is moderately synchronized in vivo [36]. Not surprisingly, artemisinin, the first line of treatment for malaria in virtually all parts of the world, also displays a life cycle dependence in its inhibition of parasite propagation [37, 38], with potency being greatest when administered early in the parasite’s life cycle. Given the continuous increase in tyrosine phosphorylation of band 3 in infected RBCs, the question naturally arose whether imatinib might similarly need to be present early in the parasite’s life cycle to prevent merozoite egress. To address this question, synchronized cultures of infected erythrocytes were treated with imatinib at different stages of their life cycle and monitored for egress and re-invasion of uninfected erythrocytes. As shown in Fig 4, the antimalarial activity of imatinib was most effective when present at the beginning of the parasite’s life cycle, with imatinib potency gradually decreasing as the parasite matured from its ring (100% suppression) to trophozoite (88% suppression) to schizont (41% suppression) stage (see also S4 Fig). Since the phosphorylation-induced weakening of the erythrocyte membrane is roughly proportional to the extent of band 3 tyrosine phosphorylation, the reduced potency of imatinib in later stage parasitized cells may derive from the fact that band 3 has already been substantially phosphorylated at these later stages of parasite development.

### Analysis of Imatinib Activity against Clinical Isolates of *P*. *falciparum* Infected Blood from Uganda

**Fig 5:**
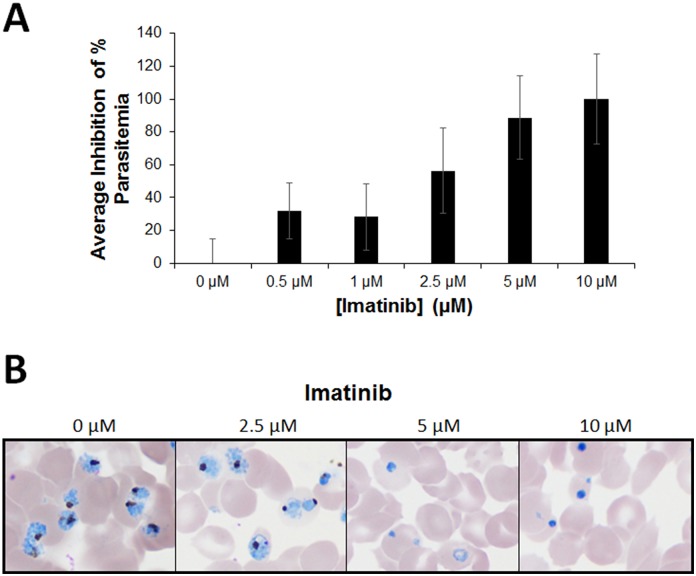
Ex vivo drug susceptibility of fresh *P*. *falciparum* clinical isolates in Uganda to imatinib treatment. Fresh blood samples from malaria patients were obtained with written informed consent and analyzed for their sensitivities to imatinib as described in Methods. (A) The parasitemia of each sample was determined as a function of imatinib concentration by qPCR analysis of extracted parasite DNA. Percent inhibition of parasitemia is plotted as a function of imatinib concentration. (B) Parasite morphology was assessed by microscopic evaluation of thin blood smears stained with fresh 10% Giemsa. Light micrographs of cultures were acquired using a Nikon Elipse CI-L microscope, Plan Fluor 100X oil objective, Numerical Aperture 1.3 at room temperature equipped with a Nikon DS-Fi2 digital camera and NIS-Elements Basic Research acquisition software. Representative images at the indicated imatinib concentrations are shown.

Since the majority of malaria infections are found in Sub-Saharan Africa [2], ex vivo studies to assess the sensitivities of fresh field isolates of *P*. *falciparum* malaria infected blood samples to imatinib were performed in Mbale, Uganda. As seen in Fig 5A, imatinib suppressed the parasitemia in these samples with an average IC_50_ of 1.37 μM (range 0.47–3.02 μM; n = 20). Surprisingly, these data suggest that imatinib has greater anti-malarial activity against random field isolates of *P*. *falciparum* than laboratory-adapted strains of the same parasite (~5 μM; see Fig 1). As shown in Fig 5B, all field isolates treated with 5 μM imatinib appeared pyknotic, with shrunken parasites containing condensed nuclei (Fig 5B); i.e. similar to the laboratory-adapted strains treated with 10 μM. These data raise the possibility that imatinib might constitute an effective treatment for *P*. *falciparum* malaria at clinically acceptable concentrations of imatinib.

**Fig 6:**
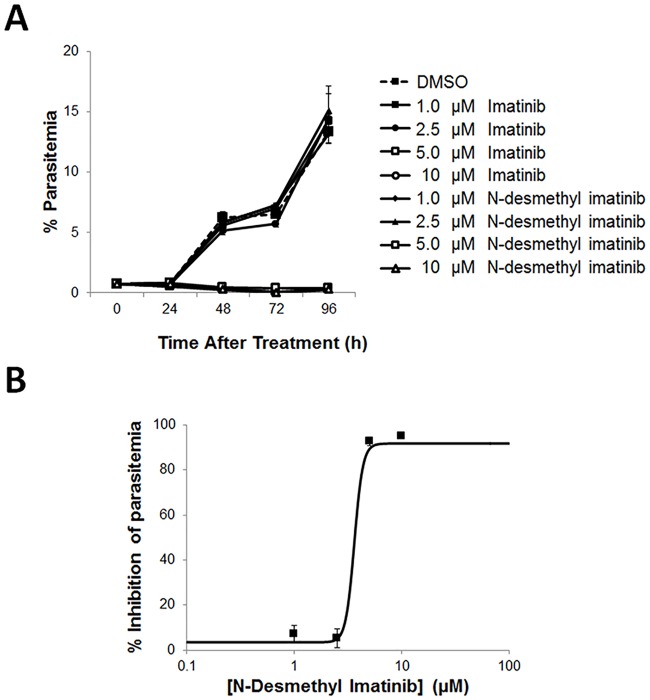
Effect of the primary imatinib metabolite in humans, N-desmethyl imatinib, on inhibition of *P*. *falciparum* parasitemia. *P*. *falciparum* strain Palo Alto cultures were treated during ring stage (0.5% parasitemia) with increasing concentrations of (A) imatinib or N-desmethyl imatinib, and % parasitemia of infected cultures was determined by flow cytometry every 24 hours after treatment to monitor parasite replication. Note, open markers inhibited all parasitemia. (B) To create a dose response curve, cultures were treated with concentrations of N-desmethyl imatinib, and the % inhibition of parasitemia was determined by flow cytometry after a 60 h incubation.

To further explore whether imatinib might serve as an effective antimalarial at concentrations known to be nontoxic in humans, an analysis of the pharmacokinetics of imatinib at an intermediate well-tolerated dose (600 mg) in cancer patients was required. Le Coutre et al. [39] report that imatinib has a clearance half-time of 17h, an average C_max_ of ~7 μM, and average C_min_ of ~2.5 μM in chronic myelogenous leukemia patients. Based on the complete inhibition of parasitemia seen in both laboratory-adapted strains and random field isolates at 5 μM (Figs 1 and 5), one might speculate that some therapeutic benefit should be seen at this same dose in malaria patients. More importantly, further scrutiny of the literature reveals that the major imatinib metabolite in humans, N-desmethyl imatinib (CGP74588), is present in patient plasma at a steady state level of ~1.4 μM [39]. Because N-desmethyl imatinib differs from the parent drug by only a single methyl group, we next decided to explore whether it might similarly exhibit anti-malarial activity. As shown in Fig 6A, N-desmethyl imatinib displays the same anti-malaria activity as imatinib (Fig 6B IC_50_ = ~3.60 μM (see also S5 Fig)), suggesting that it should probably contribute additively to suppression of the parasitemia in vivo. Taken together, these results argue that the total concentration of pharmacologically active imatinib in the serum of cancer patients should be sufficient to suppress the parasitemia in malaria patients [39–41].

## Discussion

Since the 2^nd^ century BCE, all malaria therapies have targeted malaria-encoded processes that are critical to parasite survival. Artemisinin, originally obtained by chewing the plant, *Artemisia annua*, is thought to function by promoting oxidative stress and inhibiting hemozoin formation within the infected red cell [42]. Quinine, found in the bark of the cinchona tree, accumulates in the *Plasmodium* food vacuole and inhibits the formation of hemozoin among other mechanisms [43, 44]. Synthetic quinine substitutes such as chloroquine, piperaquine, and mefloquine have subsequently been introduced to offset the resistance that has emerged to each preceding quinine congener [45], and several promising new therapies have been designed to inhibit the *P*. *falciparum* cation ATPase (PfATP4), *P*. *falciparum* protein kinase G (PfPKG), and *P*. *falciparum* phosphatidylinositol-3 kinase (PfPI3K) in the parasite [7, 46–49]. The obvious limitation of each of these approaches is that they target enzymes/processes encoded in the parasite genome, allowing the parasite to explore resistance mechanisms via constitutive mutagenesis. With many infected individuals containing >50,000,000 parasites/ml blood, the probability of selecting a resistant mutant would seem high, especially given the tendency of patients in remote regions to refrain from taking their full 3-day dosing regimen in order to save the remaining pills for a subsequent bout with the disease [50]. These observations all argue that complete eradication of malaria may require a pharmaceutical that can target a host enzyme that cannot be mutated by the parasite [36].

In this article, we describe a novel antimalarial agent that blocks parasitemia by inhibiting a process encoded in the host cell’s genome. As we have shown elsewhere, phosphorylation of band 3 on tyrosines 8 and 21 is catalyzed by syk tyrosine kinase [51], leading to a major reorganization of the RBC membrane that induces release of glyceraldehyde 3-phosphate dehydrogenase, lactate dehydrogenase, phosphofructokinase, pyruvate kinase, aldolase and deoxyhemoglobin from band 3 [52, 53]. More importantly, this phosphorylation also causes displacement of ankyrin from band 3, severing the major bridge connecting the RBC membrane to its structurally stabilizing cytoskeleton. The natural consequence of this membrane reorganization in uninfected red cells is membrane weakening, bilayer vesiculation, and erythrocyte hemolysis [11, 12, 26, 27]. The steady increase in tyrosine phosphorylation of band 3 in parasitized erythrocytes undoubtedly leads to a similar membrane weakening, contributing to the observed membrane crenations and vesiculation seen in later stage infected red cells, and ultimately to the successful egress of the parasite from the destabilized cell.

To inhibit this process, we treated the malaria infected cultures with imatinib, a tyrosine kinase inhibitor that has known activity against syk [28]. The result of this treatment was prevention of parasite egress. Although it cannot be ruled out that a *P*. *falciparum* encoded dual-specificity kinase might be capable of catalyzing the tyrosine phosphorylation of band 3, or that imatinib might inhibit this kinase or some other parasite protein and thereby block parasitemia, the likelihood that such an unknown target constitutes the site of action of imatinib seems remote, especially since the crystal structure of imatinib bound to syk shows a very high degree of structural complementarity and the remarkable specificity of imatinib for a very limited number of tyrosine kinases constitutes its source of notoriety [15, 16].

It is important to emphasize that the malaria parasite engages actively in erythrocyte membrane remodeling from the moment that it enters the red cell. Thus, novel malaria-encoded proteins are repeatedly inserted into the host cytoskeleton and plasma membrane [54, 55], while normal erythrocyte membrane proteins continually undergo parasite-induced phosphorylation and proteolysis [56, 57]. Although many of these modifications may have evolved to render the erythrocyte more hospitable to parasite maturation, some of the changes have undoubtedly emerged to weaken the red cell membrane and facilitate parasite egress at the end of its life cycle [58, 59]. It will be interesting to determine whether inhibition of any of these other parasite-encoded egress processes might synergize with inhibition of the band 3 tyrosine phosphorylation in preventing progression of the parasite through its life cycle.

It is important to note that previous work from prominent labs has provided additional motivation for seeking a malaria therapy that targets an erythrocyte-mediated process. Thus, Doerig and colleagues [60] have shown that inhibition of an erythrocyte MEK1 kinase using allosteric inhibitors has parasiticidal effects on *P*. *falciparum*, both in red cells and in hepatocytes in vitro. Millholland and coworkers [57] have similarly found that activation of host protein kinase C is responsible for initiating a signaling cascade leading to proteolysis of the host protein adducin, thus cleaving the second of the two major bridges (other than the ankyrin bridge) that anchors the red cell membrane to its underlying cytoskeleton; i.e. further weakening the erythrocyte membrane. Along the same lines, this activation of protein kinase C or casein kinase is also found to lead to phosphorylation of protein 4.1, causing disruption of the spectrin-actin interaction and thereby contributing to the aforementioned membrane destabilization [61, 62]. In a similar manner, Murphy et al. [63] have demonstrated that *P*. *falciparum* co-opts an erythrocyte Gs protein to activate signaling pathways required for intra-erythrocytic parasite growth and invasion. Finally, Brizuela et al. [64] have reported that the parasite utilizes a red cell antioxidant protein, peroxiredoxin 2, to compensate for its lack of catalase and inability to rapidly detoxify hydrogen peroxide. When considered together, these observations suggest that a wide variety of erythrocyte pathways can potentially be targeted to develop a more mutation resistant therapy for malaria.

In conclusion, we suggest that imatinib constitutes an excellent candidate for clinical evaluation as an anti-malaria therapy for several reasons: i) it is FDA approved for use in both adults and children, ii) it is generally non-toxic and can be taken daily in perpetuity, iii) well tolerated concentrations demonstrating complete antimalarial activity can be achieved in patient’s plasma, iv) imatinib has been well studied in nearly all human populations, and iv) imatinib’s mechanism of action renders development of resistance mutations unlikely. With the focus of the malaria field on the design of mutation-resistant drug cocktails that might collectively enable eradication of parasitemia with a single pill, the contribution of a drug like imatinib could prove valuable.

## Materials and Methods

### Parasites and Compounds

The *Plasmodium falciparum* strains used in these studies, Dd2 (MRA-156) and FUP Palo Alto (MRA -915), were obtained from Malaria Research and Reference Reagent Resource Center (MR4). The protein tyrosine kinase inhibitors bafetinib, PP121, R406, PRT062607, dasatinib, and nilotinib were purchased from Selleck Chemicals. Imatinib mesylate and N-desmethyl imatinib were from AlsaChim.

### *P*. *falciparum* Culture and Synchronization

Fresh blood was collected from healthy adult volunteers into acid-citrate-dextrose (ACD) glass vacutainer tubes (BD Biosciences) and prepared as previously described [11]. Written informed consent was collected prior to blood donation. This study was approved by the Purdue University Institutional Review Board and conducted in accordance with Good Clinical Practice guidelines and the Declaration of Helsinki. Briefly, RBCs were separated from plasma and leukocytes by three washings in wash media (RPMI 1640 (Invitrogen) media containing 2mM glutamine, 25mM HEPES, 20mM glucose, 27μg/mL hypoxanthine and 32μg/mL of gentamicin (Sigma) (pH 7.2)). *P*. *falciparum* strains Palo Alto and Dd2 were then cultured at 1–5% hematocrit, as previously described but with minor modifications [65]. Parasites were maintained under a 1% O_2_, 5% CO_2_, and 94% N_2_ atmosphere in complete media (wash media supplemented with 0.5% Albumax II (Gibco)). Parasites were synchronized by isolating late stage parasites on a Percoll density gradient (Sigma), washing 3x to remove the Percoll, and adding the schizont stage parasitized cells to fresh RBC cultures. After 4 h incubation to allow for re-invasion of fresh RBCs, cultures were treated with aqueous 5% sorbitol for 5 min at room temperature to lyse any residual late stage parasitized cells, preserving primarily ring stage parasites and uninfected RBCs [66, 67]. After allowing the synchronized parasites to mature and reinvade fresh RBCs, therapeutic studies with the listed pharmacological agents at the desired time in hours post-invasion (hpi). To assess parasitemia and infected cell morphology, thin smears were prepared, labeled with Diff-Quick stain (Siemens), and examined by light microscopy.

### Drug Susceptibility Assays of Cultured Parasites

Synchronous ring stage *P*. *falciparum* Palo Alto or Dd2 strain infected erythrocytes were treated with the above tyrosine kinase inhibitors at 2% hematocrit and 0.5–2% parasitemia. All inhibitors were solubilized in anhydrous DMSO at a 10mM stock concentration and serially diluted in anhydrous DMSO prior to addition to malaria cultures. Untreated cultures were run in parallel with the same final concentration of DMSO as the drug treated cultures. Parasitemia was measured by flow cytometry at the desired time points and thin smears were prepared for evaluation of parasite integrity and morphology by light microscopy (see below).

### Analysis of Parasite Number and Morphology by Flow Cytometry and Light Microscopy

Malaria infected erythrocyte cultures were stained with SYBR Green I DNA stain (1:10,000 final concentration (Invitrogen); excitation wavelength, 488nm) to quantitate residual percent parasitemia using a Becton Dickinson FACSCalibur flow cytometer according to well defined procedures [68, 69]. Briefly, after 20-min incubation of the treated cultures in SYBR Green at room temperature, the cells were washed 3X in PBS-glucose [PBS containing 5mM glucose (pH 7.4)] and analyzed by flow cytometry. SYBR Green positive cells were assumed to be infected cells, since all nonerythroid cells were previously removed during erythrocyte washings and Percoll gradients. For each sample, 200,000 events were acquired using FL-1 channel and analyzed using FlowJo software (Tree Star Inc.). Blood smears from each sample were also prepared, fixed with methanol, stained in fresh 3.5% Giemsa modified stain solution (Fluka, Sigma) for 10 minutes, and then assessed by light microscopy to evaluate parasite morphology and integrity.

### Analysis of Band 3 Tyrosine Phosphorylation at Different Stages of Malaria Maturation

*P*. *falciparum-*infected cells at 20% parasitemia and the desired stage of parasite maturation were incubated for 24h in the presence of different concentrations of imatinib (0, 1 and 5μM) and then lysed at 0°C in hemolysis buffer (5 mmol/L sodium phosphate, 1 mmol/L EDTA, pH 8.0) containing protease and phosphatase inhibitor cocktails (Sigma- Aldrich, St. Louis, MO, USA). Erythrocyte membranes were prepared according to standard procedures [70] and stored at -20°C until analysis. Isolated membranes were then solubilized in Laemmli Buffer and loaded on to 8% polyacrylamide gels for SDS-PAGE. Proteins were transferred to nitrocellulose membranes, probed with mouse anti-phosphotyrosine antibody ((Santa Cruz Biotechnology) diluted 1:1000, and visualized with anti-mouse secondary antibody conjugated to IR800CW fluorescent dye (Li-COR-USA). Detection was performed using a 700–800 nm laser scanner (Odissey, Licor, USA).

### Ex vivo Sensitivity Testing of Clinical Isolates and quantification of parasite DNA by qPCR

Blood samples were acquired with written informed consent from *P*. *falciparum-*infected patients at the Mbale Regional Referral Hospital, Mbale, Uganda, following approval from the hospital’s IRB and from the Uganda National Council for Science and Technology. Research was conducted according to the principles expressed in the Declaration of Helsinki. Infected erythrocytes were isolated by three washings in wash medium and resuspended at 2% hct in wash media supplemented with 10% heat-inactivated human serum. Infected cultures were added to culture plates pre-dosed with DMSO, dihydroartemisinin, or imatinib concentrations in triplicate and maintained under a reduced oxygen atmosphere at 37°C. After a 50 h incubation, 10 μl of the infected blood cell suspension were spotted onto filter paper in triplicate and dried at room temperature for 20 minutes or stored at -20°C until use. Malaria parasite DNA was extracted according to standard procedures [71] and DNA amplification was performed using primers and probes for the 18S rRNA gene of *P*. *falciparum* as previously described with modifications [72]. Briefly parasite DNA was amplified in reaction buffer (1.5 mM MgCl2, 0.2 mM deoxynucleoside triphosphate, 300 nM of each desired primer, 100 nM of the Taqman probe and 1U of Platinum Taq polymerase (Life technologies, CA, U.S.A)). Amplification and detection of purified parasite DNA were performed using CFX96 Touch^™^ Real-Time PCR Detection System (Biorad) using the following program: 95°C for 3 min, followed by 45 cycles at 95°C for 15 min and 60°C for 45 min. Samples were run in duplicate. Percent parasitemia was estimated using a standard curve obtained from serial dilutions of *P*. *falciparum* DNA in cultures with known parasitemia using CFX Manager^™^ Software 3.1 (Biorad). The 50% inhibitory concentrations (IC_50_) were calculated by nonlinear regression analysis using ICEstimator software 1.2 [73] and by counting percent parasitemia in thin blood smears stained with 10% fresh Giemsa.

## Supporting Information

S1 Fig Effect of the tyrosine kinase inhibitors on inhibition of *P*. *falciparum* parasitemia. Synchronous P. falciparum strain Palo Alto cultures were treated during ring stage (0.5% parasitemia) with increasing concentrations of bafetinib, PP-121, imatinib, PRT062607. After 60 h incubation, % parasitemia of infected cultures was determined by flow cytometry. Results were obtained with each concentration examined in triplicate. Error bars are within the size of the point marker.
(TIF)

S2 Fig Time course effect on parasitemia of *P*. *falciparum* cultures treated with tyrosine kinase inhibitors. Synchronous P. falciparum strain Palo Alto cultures were treated during ring stage (0.5% parasitemia) with increasing concentrations of bafetinib, dasatinib, imatinib, nilotinib, PP-121, PRT062607. Infected cultures were analyzed every 24 hours to determine % parasitemia by flow cytometry. Results were obtained with each concentration examined in triplicate.
(TIF)

S3 Fig Time course effect on parasitemia of *P*. *falciparum* cultures treated with tyrosine kinase inhibitors. Synchronous P. falciparum strain Dd2 cultures were treated during the late ring stage (2% parasitemia) with increasing concentrations of bafetinib, dasatinib, R406, gefitinib, imatinib, nilotinib, PP-121, PRT062607. Infected cultures were analyzed every 11–24 hours to determine % parasitemia by flow cytometry. Results were obtained with each concentration examined in triplicate.
(TIF)

S4 Fig Time course of % parasitemia in the multiple developmental stages cultured with 0 or 8 μM of imatinib. Imatinib (8 μM) was added to synchronized *P*. *falciparum* (Dd2 strain) cultures at different stages of their life cycle, ring stage ~7hpi, late ring stage ~14hpi, trophozoite stage ~21 hpi, late trophozoite stage ~28hpi, and schizont stage ~36hpi. Parasitemia was measured by flow cytometry every 11 hours after treatment until the untreated parasites had progressed 24 h into their second infective cycle. Results were obtained with each concentration examined in triplicate.
(TIF)

S5 Fig Time course comparison of % parasitemia in imatinib and N-desmethyl imatinib treated cultures. Synchronized ring stage (12hpi) P. falciparum (Palo Alto strain) cultures at 0.75% parasitemia were treated with the indicated concentrations N-desmethyl imatinib. After 60 h incubation, % parasitemia of infected cultures was determined by flow cytometry. Results were obtained with each concentration examined in triplicate.
(TIF)

S1 Table Effect of different concentrations of imatinib on parasite growth rate during the first 12h of each life cycle. Conditions are as shown in Fig 1B. The percent parasite growth per hour was calculated from the percent increase in parasitemia over the first 12h of each life cycle.
(TIF)
