# Supplementary figures and images for: Inhibition of an Erythrocyte Tyrosine Kinase with Imatinib Prevents Plasmodium falciparum Egress and Terminates Parasitemia
Source: PLoS One. 2016 Oct 21;11(10):e0164895. doi: 10.1371/journal.pone.0164895 (PMC5074466; doi:10.1371/journal.pone.0164895)

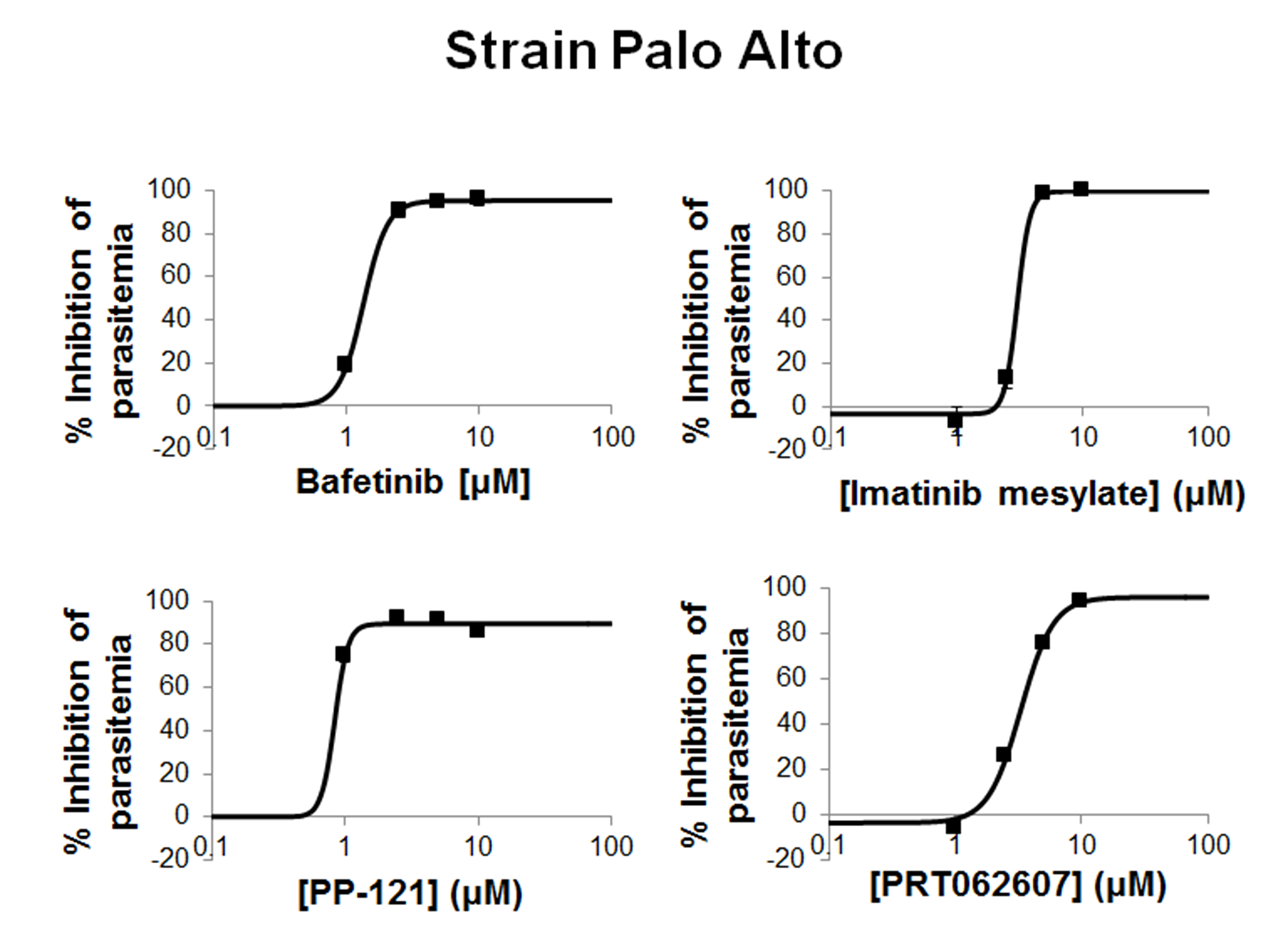

Supplement: S1 Fig — Synchronous P. falciparum strain Palo Alto cultures were treated during ring stage (0.5% parasitemia) with increasing concentrations of bafetinib, PP-121, imatinib, PRT062607. After 60 h incubation, % parasitemia of infected cultures was determined by flow cytometry. Results were obtained with each concentration examined in triplicate. Error bars are within the size of the point marker. (TIF) [file pone.0164895.s001.tif]

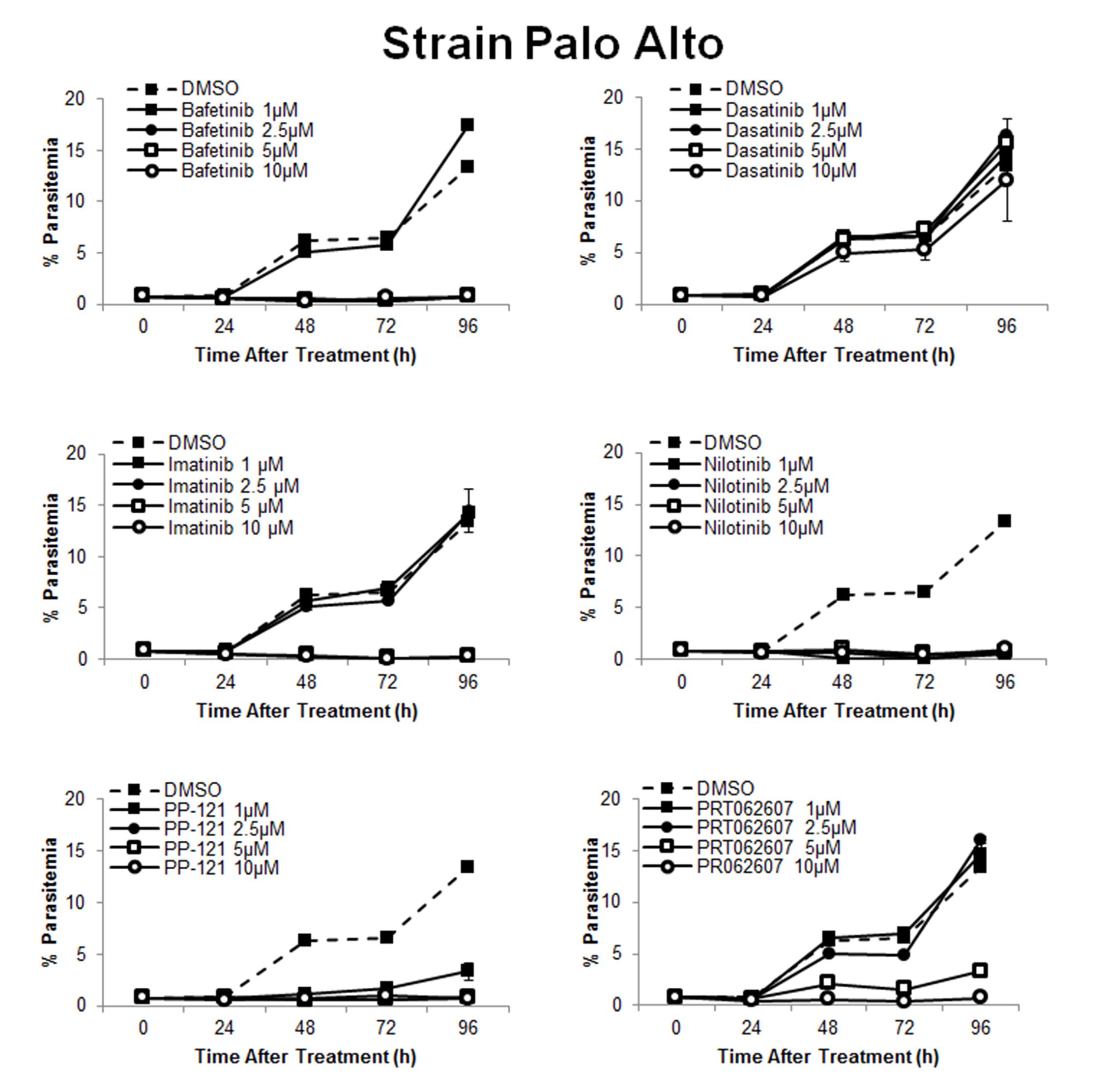

Supplement: S2 Fig — Synchronous P. falciparum strain Palo Alto cultures were treated during ring stage (0.5% parasitemia) with increasing concentrations of bafetinib, dasatinib, imatinib, nilotinib, PP-121, PRT062607. Infected cultures were analyzed every 24 hours to determine % parasitemia by flow cytometry. Results were obtained with each concentration examined in triplicate. (TIF) [file pone.0164895.s002.tif]

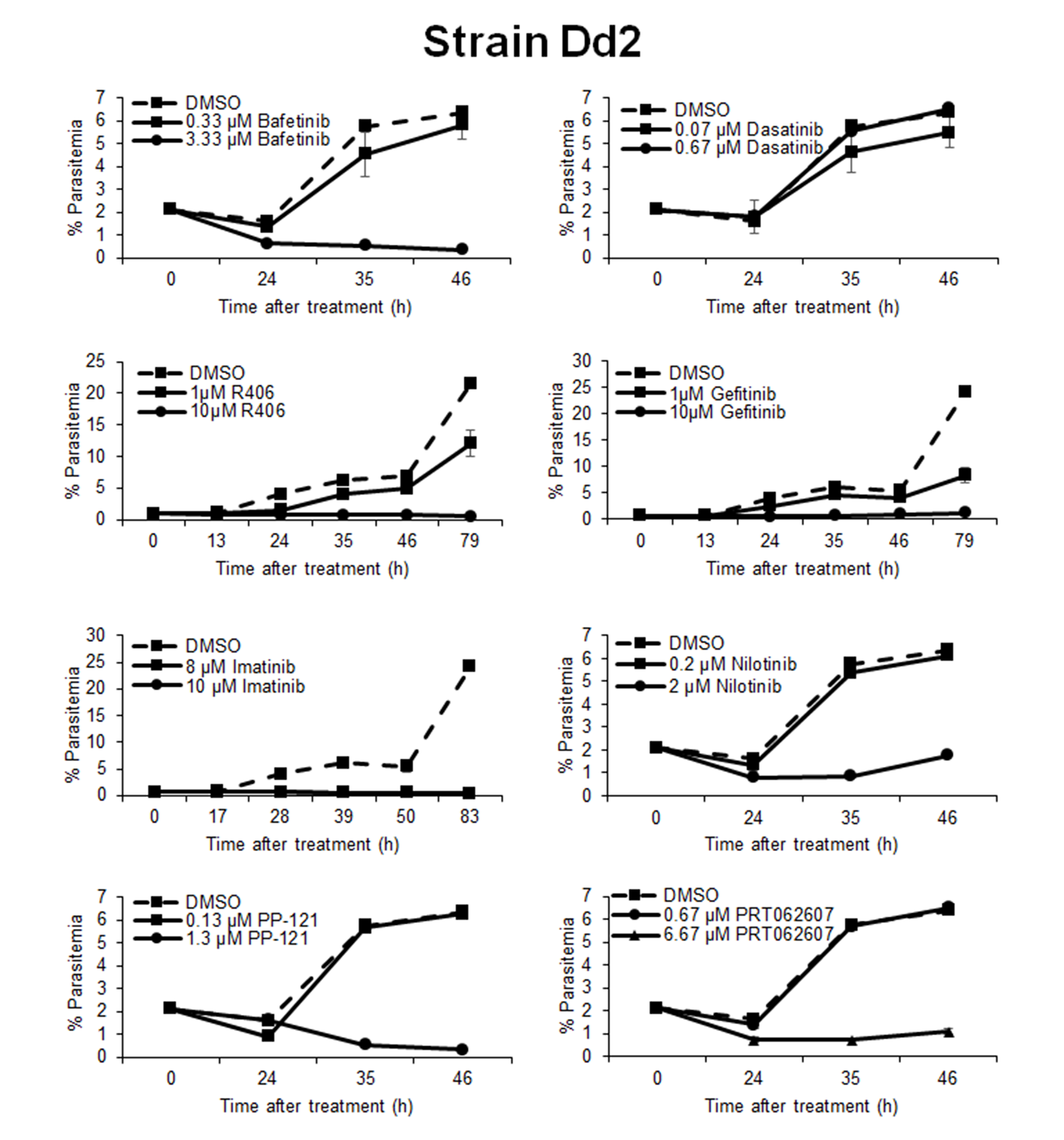

Supplement: S3 Fig — Synchronous P. falciparum strain Dd2 cultures were treated during the late ring stage (2% parasitemia) with increasing concentrations of bafetinib, dasatinib, R406, gefitinib, imatinib, nilotinib, PP-121, PRT062607. Infected cultures were analyzed every 11–24 hours to determine % parasitemia by flow cytometry. Results were obtained with each concentration examined in triplicate. (TIF) [file pone.0164895.s003.tif]

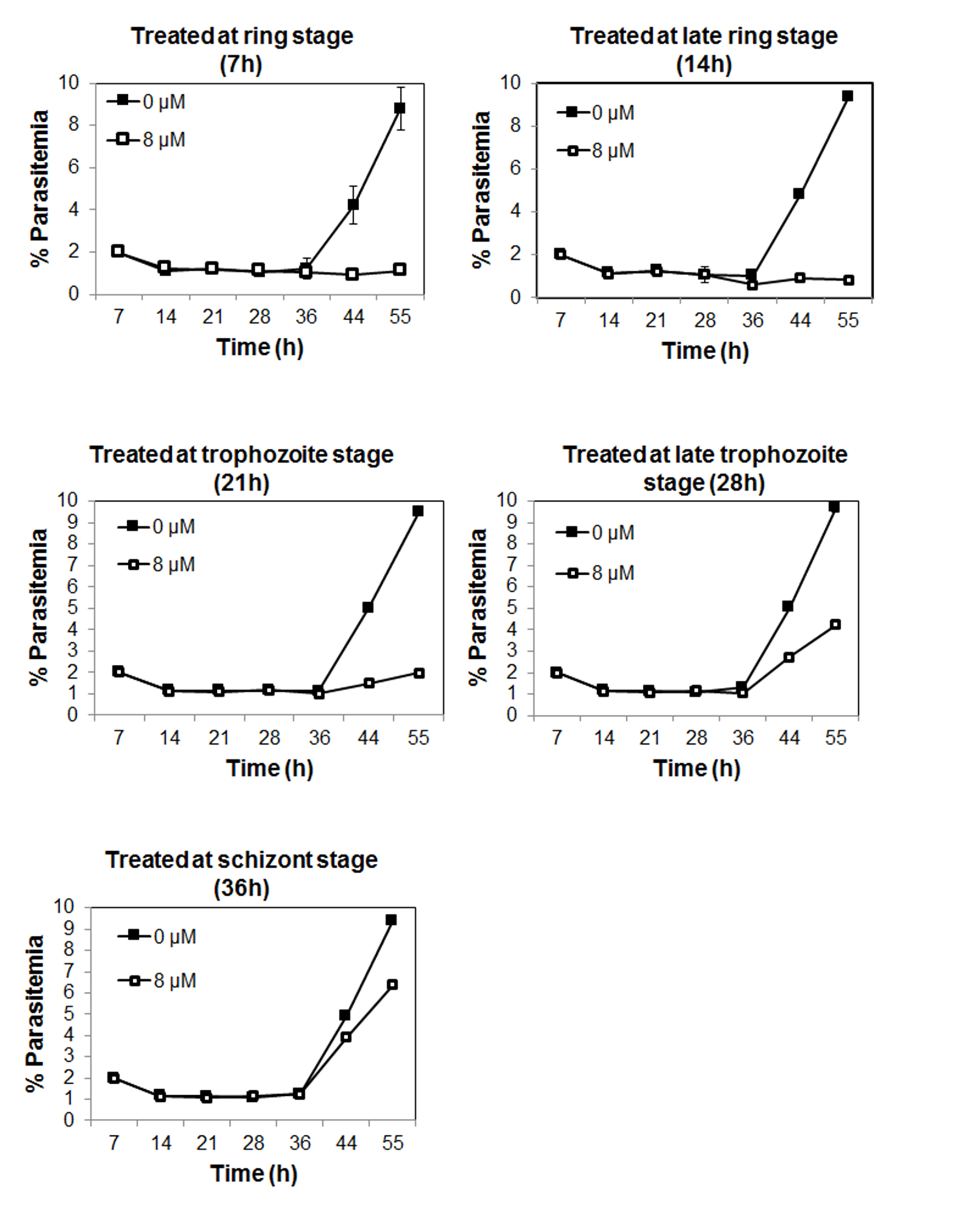

Supplement: S4 Fig — Imatinib (8 μM) was added to synchronized P. falciparum (Dd2 strain) cultures at different stages of their life cycle, ring stage ~7hpi, late ring stage ~14hpi, trophozoite stage ~21 hpi, late trophozoite stage ~28hpi, and schizont stage ~36hpi. Parasitemia was measured by flow cytometry every 11 hours after treatment until the untreated parasites had progressed 24 h into their second infective cycle. Results were obtained with each concentration examined in triplicate. (TIF) [file pone.0164895.s004.tif]

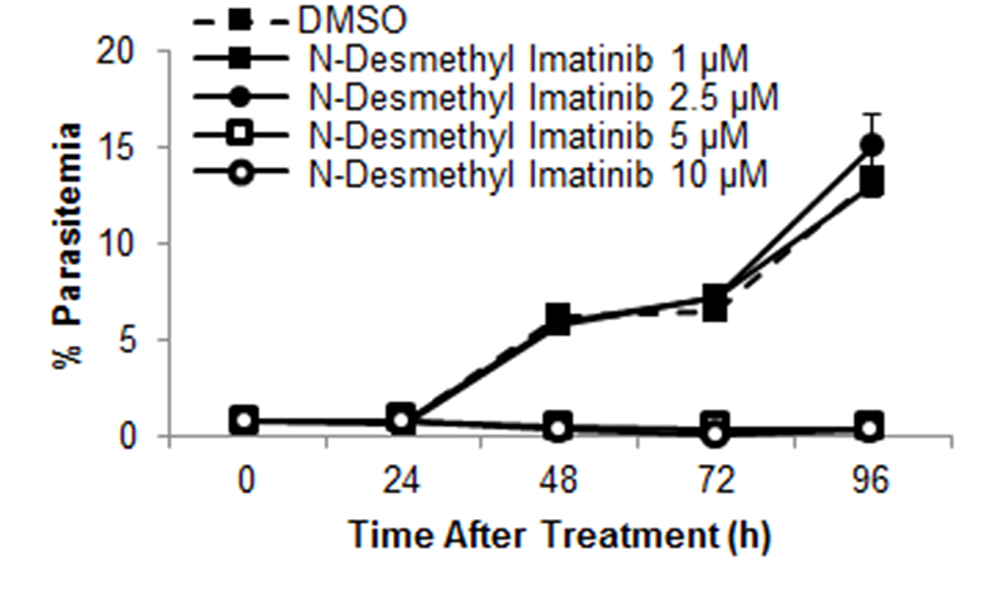

Supplement: S5 Fig — Synchronized ring stage (12hpi) P. falciparum (Palo Alto strain) cultures at 0.75% parasitemia were treated with the indicated concentrations N-desmethyl imatinib. After 60 h incubation, % parasitemia of infected cultures was determined by flow cytometry. Results were obtained with each concentration examined in triplicate. (TIF) [file pone.0164895.s005.tif]

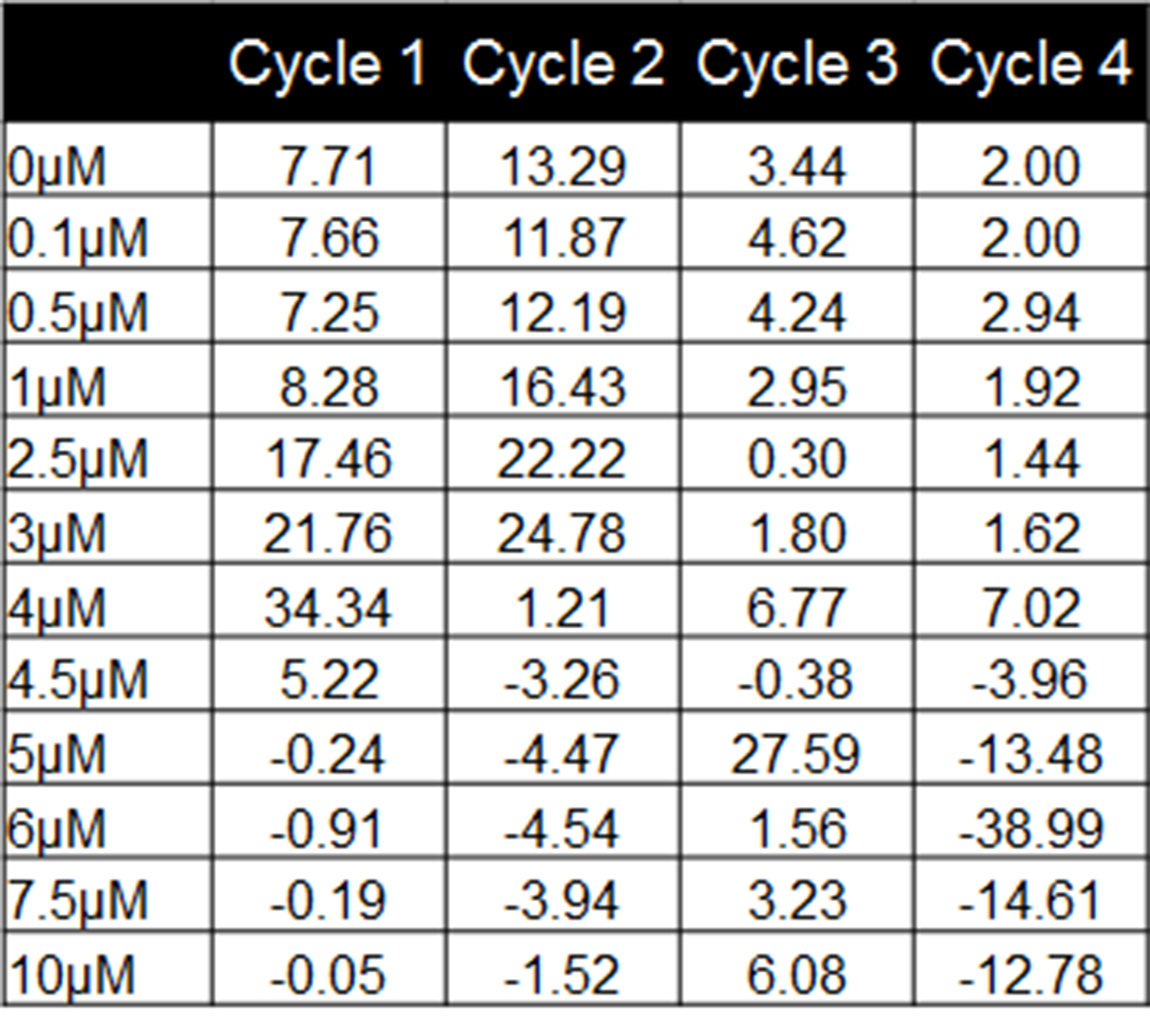

Supplement: S1 Table — Conditions are as shown in Fig 1B. The percent parasite growth per hour was calculated from the percent increase in parasitemia over the first 12h of each life cycle. (TIF) [file pone.0164895.s006.tif]
